# Biocontrol of Fusarium Species Utilizing Indigenous Rooibos and Honeybush Extracts

**DOI:** 10.1128/spectrum.02742-22

**Published:** 2023-05-24

**Authors:** Beauty E. Omoruyi, Heinrich Volschenk, Wentzel C. A. Gelderblom, Mariska Lilly

**Affiliations:** a Applied Microbial and Health Biotechnology Institute, Cape Peninsula University of Technology, Cape Town, South Africa; b Department of Microbiology, Stellenbosch University, Stellenbosch, South Africa; Huazhong Agricultural University

**Keywords:** rooibos, honeybush, *Fusarium* species, antifungal activity

## Abstract

Mycotoxins produced by several Fusarium species have a significant effect on reducing maize yield and grain quality and have led to food safety concerns. The antifungal activities of rooibos (Aspalathus linearis) and honeybush (*Cyclopia* species) tea extracts reduced the growth of plant pathogen Botrytis cinerea, but their efficacy against Fusarium spp. is unknown. In this study, we examined the effects of fermented and unfermented rooibos (*A. linearis*) and honeybush (Cyclopia subternata) aqueous extracts as well as green tea (Camellia sinensis) against 10 Fusarium species. Conidial viability was assessed by fluorescence microscopy dyes, ATP production was determined using the BacTiter-Glo assay, the mode of action was analyzed by scanning electron microscopy (SEM), and quantification of polyphenols was done using high-performance liquid chromatography with diode array detection (HPLC-DAD). Fermented rooibos extract demonstrated the highest antifungal activity (*P* < 0.0001) against Fusarium verticillioides MRC 826-E, Fusarium subglutinans MRC 8553, Fusarium proliferatum MRC 8549, and Fusarium globosum MRC 6647, with only 9.53%, 9.26%, 11.0%, and 12.7% ATP production, respectively, followed by antifungal activity of the fermented *C. subternata* extract against *F. subglutinans* MRC 8553, *F. subglutinans* MRC 8554, F. proliferatum MRC 8550, and F. verticillioides MRC 826-E with 3.79%, 6.04%, 6.04%, and 8.40% ATP production, respectively. Extract-treated conidia examined by SEM exhibited disruption of conidial hyphae and collapsed spores. Overall, the fermented rooibos and *C. subternata* extracts showed higher antifungal activity against the Fusarium species than the unfermented extracts.

**IMPORTANCE** In maize subsistence farming areas in South Africa, daily consumption of maize contaminated by high level of mycotoxins contributes to long-term health effects such as immune deficiency and cancer. Biocontrol methods that are safe and cost-effective are critical to addressing this public health problem. Plant extracts known as biocides or green pesticides are alternatives to chemical pesticides due to their safety and eco-friendly properties. In South Africa, rooibos (Aspalathus linearis) and honeybush (*Cyclopia* species) contain polyphenols with significant antioxidant and antimicrobial properties. These indigenous herbal teas are widely available and consumed in South Africa and have potential as an innovative approach to reduce mycotoxin levels and, subsequently, human and animal exposure to these toxins. This study evaluates the efficacy of the antifungal activities of several aqueous extracts prepared from fermented and unfermented rooibos (*A. linearis*), honeybush (Cyclopia subternata), and green tea (Camellia sinensis) on 10 Fusarium strains.

## INTRODUCTION

Food and feed are subject to infection by a variety of toxigenic microorganisms capable of causing foodborne diseases in animals and humans worldwide (1). Fungi produce toxic secondary metabolites (mycotoxins) in commodities such as maize, wheat, soybean, and rice, resulting in ear rot, head blight, stem rot, and loss of nutritional components during storage (2). Several Fusarium species produce mycotoxins in maize with major infections, including Fusarium verticillioides, Fusarium graminearum, Fusarium subglutinans, Fusarium proliferatum, and Fusarium globosum (3, 4). The prevalence of F. graminearum species (FGS) is associated with mycotoxin type B trichothecenes (TCT-B), zearalenone (ZEA), nivalenol (NIV), and acetylated derivatives (15-ADON and 3-ADON) in wheat, rice, barley, rye, and oats (5). F. verticillioides, F. proliferatum, *F. subglutinans*, and *F. globosum* produce fumonisins (FB_1_, FB_2_, and FB_3_), especially in maize and sorghum. Higher concentrations of these mycotoxins in animal feed exert toxic effects in farm animals, causing distress and reduced productivity (6). Acute intoxication with fumonisins in domestic animals has resulted in pulmonary edema in pigs and dogs, leukoencephalomalacia in horses, and brain hemorrhage and kidney and liver cancer in rabbits (7). To prevent the negative effects on animals and consumers, many countries regulate mycotoxin concentrations in feed. Common symptoms associated with consuming mycotoxin-contaminated products include frequent vomiting, diarrhea, drowsiness, anemia, eye infection, skin infection, nail infection, and esophageal cancer, as well as stunting in children. High levels of fumonisin intake in pregnant women could also result in neural tube defects in the fetus (8). In the European Union (EU), for example, maximum levels are enforced for FB_1_ (9), and guidance values have been stipulated for fumonisins, ZEA, and deoxynivalenol (DON) (10). Therefore, mycotoxin concentrations in feed and food should be continuously monitored to support risk assessment.

Current fungal pesticides such as ammonia detoxification, sodium hydroxide, and sulfur dioxide have led to serious environmental (soil contamination), food security (decreased nutritional value of foods and sensory quality) and human health (toxic derivatives) concerns (11). Due to the harmful effects of chemicals, a safe, cost-efficient alternative biocotrol approach is critical to addressing the public health problem. Plant extracts known as biocides or green pesticides are deemed sustainable alternatives due to their safety and eco-friendly properties. Plant-derived compounds for disease management can provide food safety and security to reduce Fusarium infections and their associated mycotoxin production in different grains produced in South Africa and beyond (12).

In South Africa, the herbal teas rooibos (Aspalathus linearis) and honeybush (*Cyclopia* species) contain polyphenols with significant antioxidant and antimicrobial properties. It has been shown that aspalathin, dihydrochalcones, flavonoids, proanthocyanidin-type polyphenols, xanthone, mangiferin, and hesperidin inhibited the growth of Escherichia coli, Streptococcus pyogenes, Staphylococcus aureus, and Candida albicans (13). The antifungal susceptibility of Botrytis cinerea spore germination by rooibos and honeybush could be linked directly to polyphenols that synergistically inhibited conidial growth (14). These indigenous herbal teas are widely available and consumed daily by the people of South Africa. They have the potential as an innovative approach to reduce mycotoxin levels and human and animal exposure. Therefore, development of a cost-effective food-grade biocontrol method from indigenous South African rooibos and honeybush tea extracts for application in subsistence farming and commercial systems against Fusarium infection and mycotoxin production will contribute to sustainable mycotoxin risk management practices in maize production.

The efficacy of extracts from *A. linearis* and *Cyclopia* spp. as antifungal agents against Fusarium species has not been previously reported. Hence, this study evaluates the effect of aqueous extracts prepared from fermented and unfermented rooibos, honeybush, and green tea on the growth inhibition of 10 Fusarium strains and thereby indirectly contributes to the reduction in mycotoxin production.

## RESULTS

### Quantification of polyphenols using HPLC-DAD.

The polyphenolic composition of the tea extracts was determined using high-performance liquid chromatography with diode array detection (HPLC-DAD) analysis (15). The polyphenol composition of rooibos differs from those of honeybush and green tea extracts. Eleven compounds were detected in the fermented and unfermented rooibos extracts, with five compounds unique to the fermented rooibos extract, including the C-C-linked β-d-glucopyranosides such as the *S*-eriodictyol-6-glucoside, *S*-eriodictyol-8-glucoside, and phenylpyruvic acid-2-*O*-glucoside (Table 1). Aspalathin, nothofagin, isoorientin, orientin, bioquercetin, hyperoside, rutin, isoquercitrin, luteoloside, vitexin, and isovitexin were detected in both the unfermented and fermented rooibos extracts, with aspalathin, nothofagin, isoorientin, and orientin detected at higher concentrations in the unfermented rooibos extract than in the fermented extract. The total polyphenols were higher in the unfermented (18.883 g/100 g extract) rooibos extract than in the fermented (7.731 g/100 g extract) rooibos extract. Major compounds detected in the honeybush extracts included benzophenones (iriflophenone-3-C-glucoside-4-O-glucoside, maclurin-3-C-glucoside), an ihydroxybenzen (protocatechuic acid), dihydrochalcones (3-hydroxyphloretin-3′,5′-di-C-glucoside, phloretin-3′,5′-di-C-glucoside), flavanones (eriocitrin and hesperidin), xanthone (mangiferin and isomangiferin), a flavonoid glycoside (vicenin-2), a hydroxycinnamic acid (*p*-coumaric acid), and a flavone (scolymoside). The unfermented honeybush extract also contained a higher total polyphenol concentration (9.105 g/100 g extract) than what was measured in the fermented honeybush extract (4.333 g/100 g extract). Caffeine content was very high in the green tea extract, followed by epigallocatechins.

**TABLE 1:**
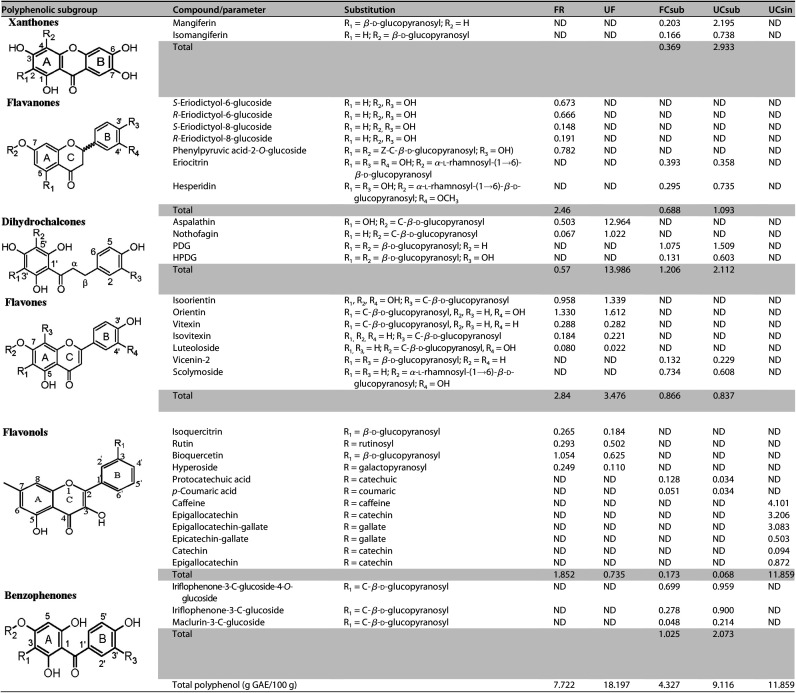
Quantification of the phenolic contents of the rooibos (*A. linearis*), honeybush (*C. subternata*), and green tea (C. sinensis) extracts by HPLC-DAD^*a*^

a Values represent a duplicate analysis with grams per 100 grams of extract determined using the Folin-Ciocalteu reagent expressed in grams per gallic acid equivalent per 100 grams of extract. PDG, phloretin-3′,5′-di-C-glucoside; HPDG, 3′,5′-di-β-d-glucopyranosyl-3-hydroxyphloretin; ND, not detected; FR and UR, fermented and unfermented rooibos extracts; FCsub and UCsub, fermented and unfermented *C. subternata* extracts, respectively; UCsin, unfermented C. sinensis extract.

### Determination of Fusarium conidial viability.

Conidial viability was determined in order to confirm the functionality of the spores to be utilized for the antifungal activities of the tea extracts on growth inhibition. Propidium iodide (PI) penetrates the nonviable conidia and stains red fluorescence, while Hoechst stain passively enters through the cell membrane, where intracellular esterases subsequently hydrolyze it to blue fluorescence. This double staining enabled differentiation between nonviable and viable fungal conidia. Viable conidia retained the Hoechst 33342 blue fluorescence (data not shown), and very few nonviable conidia were visible with the red fluorescent staining patterns of the PI dye.

### Determination of the antifungal activity of herbal tea extracts.

**FIG 1:**
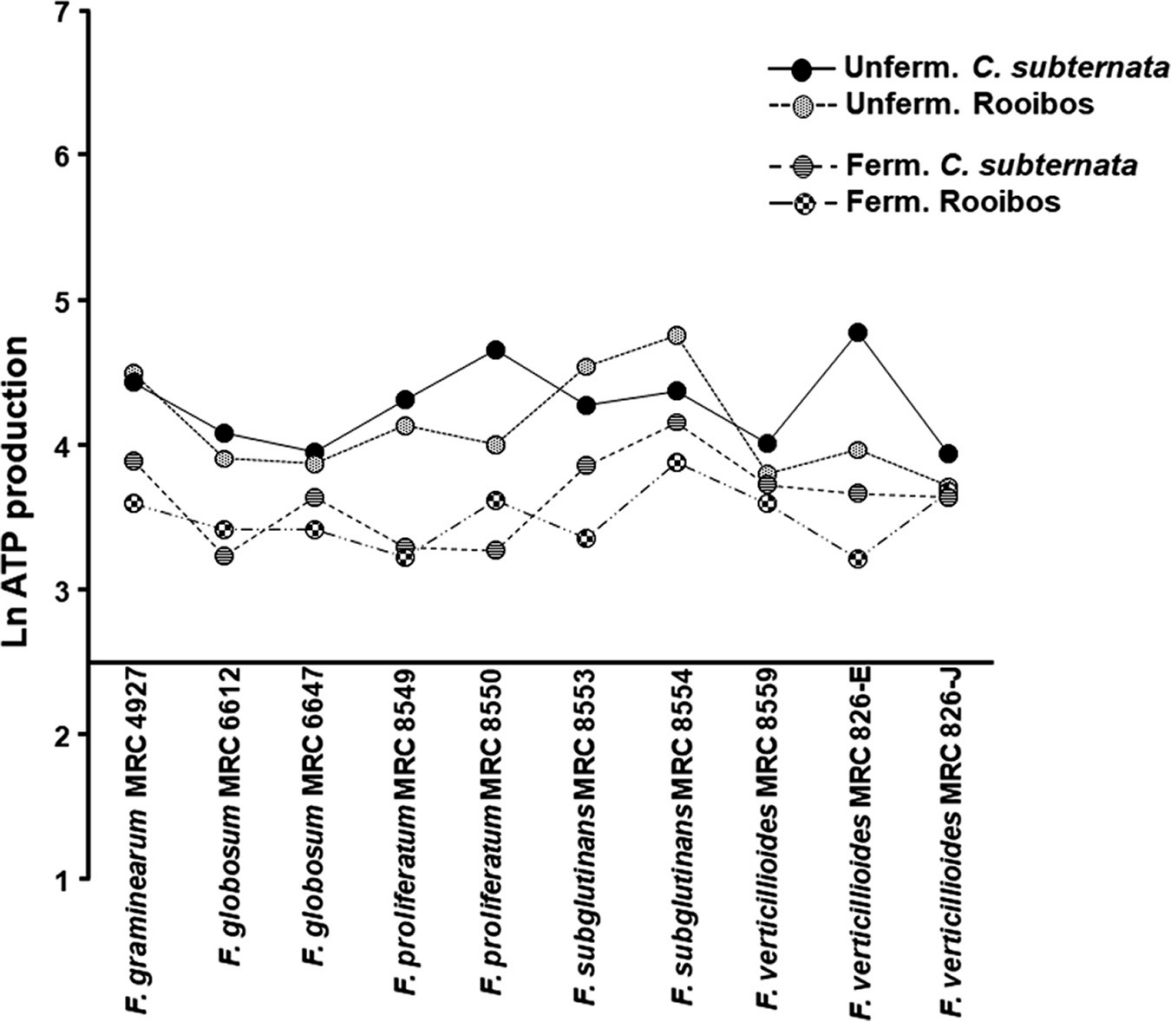
Natural log (Ln) of ATP production of 10 Fusarium strains treated with tea extracts, with fermented rooibos showing the most effective antifungal activity, followed by fermented *C. subternata*, then unfermented rooibos, then unfermented *C. subternata*.

The efficacy of the tea extracts on Fusarium conidia growth inhibition was determined by measuring the percentage of ATP using different tea extract concentrations. The fermented rooibos tea extract at 20 mg/mL reduced conidial viability of F. verticillioides MRC 826-E, *F. subglutinans* MRC 8553, and F. proliferatum MRC 8549 to 9.53% (50% inhibitory concentration [IC_50_], 1.19 ± 0.05), 9.26% (IC_50_, 1.79 ± 0.05), and 11.0% (IC_50_, 0.77 ± 0.04) of their respective controls after 96 h of incubation (Table 2 and Fig. 1). The 20-mg/mL treatment with unfermented rooibos tea extract reduced the conidial viability of F. verticillioides MRC 826-E, *F. subglutinans* MRC 8553, and F. proliferatum MRC 8549 to 44.4% (IC_50_, 4.97 ± 0.31), 29.5% (IC_50_, 12.56 ± 0.37), and 23.6% (IC_50_, 8.26 ± 0.41) ATP production, respectively, compared to the control treatments. However, 20 mg/mL of unfermented rooibos tea extract showed the highest growth inhibition against F. verticillioides MRC 8559, F. verticillioides MRC 826-J, and *F. globosum* MRC 6647, reducing conidial viability to 18.0% (IC_50_, 3.81 ± 0.07), 22.6% (IC_50_, 2.21 ± 0.17), and 22.9% (IC_50_, 4.01 ± 0.17) ATP production, respectively (Table 3). The extracts prepared from the fermented honeybush tea demonstrated higher antifungal potency than the unfermented honeybush extract at the highest concentration (20 mg/mL) (Table 4 and Table 5). Among the 10 Fusarium strains, five strains, including *F. subglutinans* MRC 8553, *F. subglutinans* MRC 8554, F. proliferatum MRC 8550, F. verticillioides MRC 826-E, and F. proliferatum MRC 8549, were highly susceptible to the fermented honeybush (Cyclopia subternata) tea, at conidial viability rates of only 3.79% (IC_50_, 6.63 ± 0.06), 6.04% (IC_50_, 9.47 ± 0.13), 6.04% (IC_50_, 1.75 ± 0.21), 8.40% (IC_50_, 4.28 ± 0.31), and 9.73% (IC_50_, 1.20 ± 0.05) ATP production, respectively, to the untreated conidia (Table 3). The unfermented *C. subternata* extract exhibited lower inhibitory effects on the same conidial strains at 44.9% (IC_50_, 10.83 ± 0.47), 34.6% (IC_50_, 13.87 ± 0.39), 87.1% (IC_50_, 45.88 ± 11.36), 76.7% (IC_50_, 32.95 ± 0.61), and 24.9% (IC_50_, 11.33 ± 0.45), respectively (Table 5). The susceptibilities of F. verticillioides MRC 826-J and *F. globosum* MRC 6647 at 30.6% (IC_50_, 4.83 ± 0.33) and 28.6% (IC_50_, 4.98 ± 0.23) to the unfermented *C. subternata* extract were not significantly different from the unfermented green tea activity of Camellia sinensis at 27.5% (IC_50_, 3.67 ± 0.21) and 25.0% (IC_50_, 9.26 ± 0.65) conidial viability, respectively (Table 6).

**TABLE 2:** Conidial viability parameters after 96 h of exposure to fermented rooibos extract^*a*^

Fusarium strain	% ATP production								IC_50_ of ATP (mg/mL)
	Control	Fermented rooibos extract						AmpB (2.5 mg/mL)	
		20 mg/mL	10 mg/mL	5 mg/mL	2.5 mg/mL	1.25 mg/mL	0.62 mg/mL		
F. verticillioides									
MRC 826-E	100 ± 0.67	9.53 ± 0.31***	12.7 ± 0.60***	18.7 ± 0.36***	34.8 ± 1.03***	42.4 ± 1.12***	69.8 ± 1.01***	50.1 ± 4.3***	1.19 ± 0.05**
MRC 826-J	100 ± 1.11	23.9 ± 13.4***	27.7 ± 1.32***	37.4 ± 1.90***	49.3 ± 1.85***	52.8 ± 1.60***	59.4 ± 0.58***	23.3 ± 5.3***	1.78 ± 0.13**
MRC 8559	100 ± 1.35	17.9 ± 1.66***	23.6 ± 1.57***	25.5 ± 2.01***	37.5 ± 1.02***	67.6 ± 2.36***	82.4 ± 1.55***	2.08 ± 0.25***	2.21 ± 0.05**
*F. subglutinans*									
MRC 8554	100 ± 5.00	15.2 ± 2.61***	30.8 ± 1.52***	48.7 ± 1.10***	68.0 ± 2.67***	85.2 ± 1.47***	95.6 ± 2.81***	1.77 ± 0.3***	4.92 ± 0.03**
MRC 8553	100 ± 2.11	9.26 ± 0.70***	16.1 ± 1.48***	17.8 ± 0.99***	43.3 ± 1.14***	65.2 ± 1.70***	71.7 ± 2.33***	3.51 ± 0.1***	1.79 ± 0.05**
F. proliferatum									
MRC 8554	100 ± 3.19	21.2 ± 1.95***	28.7 ± 1.45***	35.1 ± 1.33***	43.3 ± 1.84***	47.9 ± 0.64***	58.9 ± 1.77***	44.1 ± 4.16***	1.41 ± 0.08**
MRC 8549	100 ± 3.34	11.0 ± 2.01***	18.7 ± 1.17***	21.1 ± 1.06***	31.9 ± 1.02***	35.5 ± 1.49***	51.3 ± 1.12***	45.2 ± 1.26***	0.77 ± 0.04**

F. graminearum MRC 4927	100 ± 1.95	19.9 ± 2.11***	21.7 ± 1.04***	27.6 ± 1.14***	43.9 ± 0.97***	51.8 ± 2.11***	86.9 ± 2.49***	0.21 ± 0.0***	2.09 ± 0.05**
*F. globosum*									
MRC 6647	100 ± 2.84	12.7 ± 0.35***	17.7 ± 0.21***	23.2 ± 1.91***	41.0 ± 2.21***	55.4 ± 1.90***	64.1 ± 2.52***	27.1 ± 4.20***	1.47 ± 0.03**
MRC 6122	100 ± 1.99	14.8 ± 0.37***	16.9 ± 1.34***	28.9 ± 0.69***	36.1 ± 1.54***	50.1 ± 1.02***	58.0 ± 3.90***	24.5 ± 2.74***	1.16 ± 0.12**

a Values represent means ± standard deviations of five replications from at least two experiments. % ATP production, percentage of spore viability of cell energy production; IC_50_ of ATP, MIC at 50% ATP; *** and **, statistically significant at *P* < 0.0001 and *P* < 0.001, respectively.

**TABLE 3:** Conidial viability parameters after 96 h of exposure to unfermented rooibos extract^*a*^

Fusarium strain	% ATP production								IC_50_ of ATP (mg/mL)
	Control	Unfermented rooibos extract						AmpB (2.5 mg/mL)	
		20 mg/mL	10 mg/mL	5 mg/mL	2.5 mg/mL	1.25 mg/mL	0.62 mg/mL		
F. verticillioides									
MRC 826-E	100 ± 0.67	44.4 ± 5.84***	43.2 ± 1.55***	46.9 ± 1.96***	50.0 ± 1.70***	63.4 ± 1.24***	75.7 ± 1.16***	50.1 ± 4.3***	4.97 ± 0.31**
MRC 826-J	100 ± 1.11	22.6 ± 1.67***	28.2 ± 1.63***	43.7 ± 1.27***	46.6 ± 2.53***	56.3 ± 2.19***	63.6 ± 2.62***	23.3 ± 5.3***	2.21 ± 0.17**
MRC 8559	100 ± 1.35	18.0 ± 1.47***	24.4 ± 2.59***	46.8 ± 1.37***	54.3 ± 2.08***	81.2 ± 1.59***	89.3 ± 2.93***	2.08 ± 0.25***	3.81 ± 0.07**
*F. subglutinans*									
MRC 8554	100 ± 5.00	41.9 ± 4.12***	71.4 ± 1.84***	112.1 ± 1.2***	152.5 ± 2.2***	211.2 ± 28***	224.5 ± 28***	1.77 ± 0.3***	16.81 ± 0.9**
MRC 8553	100 ± 2.11	29.5 ± 2.29***	57.5 ± 1.87***	91.3 ± 2.40***	133.9 ± 1.4***	145.9 ± 3.9***	228.1 ± 20***	3.51 ± 0.1***	12.56 ± 0.3**
F. proliferatum									
MRC 8550	100 ± 3.19	36.2 ± 1.62***	42.8 ± 1.95***	60.1 ± 2.46***	61.8 ± 1.03***	67.5 ± 2.93***	69.7 ± 1.45***	44.1 ± 4.16***	6.63 ± 0.41**
MRC 8549	100 ± 3.34	23.6 ± 1.99***	33.2 ± 1.95***	77.3 ± 2.54***	85.1 ± 3.92***	91.7 ± 1.81***	123.0 ± 1.8***	45.2 ± 1.26***	8.26 ± 0.41**

F. graminearum MRC 4927	100 ± 1.95	44.1 ± 1.22***	53.2 ± 1.84***	85.8 ± 2.40***	108.8 ± 1.7***	131.9 ± 2.2***	177.8 ± 14***	0.21 ± 0.02***	14.20 ± 0.3**
*F. globosum*									
MRC 6647	100 ± 2.84	22.9 ± 1.57***	31.9 ± 1.51***	53.1 ± 2.66***	60.5 ± 1.73***	69.7 ± 1.73***	72.3 ± 1.78***	27.1 ± 4.2***	4.01 ± 0.17**
MRC 6122	100 ± 1.99	30.0 ± 1.19***	42.6 ± 2.07***	46.4 ± 0.29***	50.1 ± 1.63***	67.9 ± 2.12***	73.6 ± 2.49***	24.5 ± 2.74***	4.11 ± 0.14**

a Values represent means ± standard deviations of five replications from at least two experiments. % ATP production, percentage of spore viability of cell energy production; IC_50_ of ATP, MIC at 50% ATP; *** and **, statistically significant at *P* < 0.0001 and *P* < 0.001, respectively.

**TABLE 4:** Conidial viability parameters after 96 h of exposure to fermented *C. subternata* extract^*a*^

Fusarium strain	% ATP production								IC_50_ of ATP (mg/mL)
	Control	Fermented *C. subternata* extract						AmpB (2.5 mg/mL)	
		20 mg/mL	10 mg/mL	5 mg/mL	2.5 mg/mL	1.25 mg/mL	0.62 mg/mL		
F. verticillioides									
MRC 826-E	100 ± 0.67	8.40 ± 1.02***	15.8 ± 1.34***	47.0 ± 4.34***	76.0 ± 2.84***	80.8 ± 4.14***	91.4 ± 1.82***	50.1 ± 4.3***	4.28 ± 0.31**
MRC 826-J	100 ± 1.11	20.6 ± 1.88***	26.6 ± 1.03**	29.9 ± 0.71***	37.8 ± 2.26***	62.2 ± 1.08***	77.5 ± 2.66***	23.3 ± 5.3***	2.11 ± 0.06**
MRC 8559	100 ± 1.35	17.2 ± 1.74***	24.3 ± 1.91***	30.5 ± 2.33***	62.7 ± 0.68***	73.0 ± 2.10***	88.2 ± 1.71***	2.08 ± 0.2***	3.37 ± 0.13**
*F. subglutinans*									
MRC 8554	100 ± 5.00	6.04 ± 0.30***	11.4 ± 0.45***	130.9 ± 3.0***	146.7 ± 7.9***	223.5 ± 20***	235.3 ± 21***	1.77 ± 0.3***	9.47 ± 0.13**
MRC 8553	100 ± 2.11	3.79 ± 0.48***	7.97 ± 0.12***	84.1 ± 0.96***	109.2 ± 2.4***	178.5 ± 23***	230.2 ± 17***	3.51 ± 0.1***	6.63 ± 0.06**
F. proliferatum									
MRC 8550	100 ± 3.19	6.04 ± 0.53***	13.5 ± 0.78***	20.7 ± 1.70***	46.8 ± 2.88***	61.3 ± 3.65***	67.9 ± 2.58***	44.1 ± 4.1***	1.75 ± 0.21**
MRC 8549	100 ± 3.34	9.73 ± 0.71***	13.7 ± 1.48***	24.0 ± 3.45***	39.8 ± 1.67***	48.4 ± 2.03***	60.9 ± 2.11***	45.2 ± 1.2***	1.20 ± 0.05**

F. graminearum MRC 4927	100 ± 1.95	20.3 ± 1.12***	42.1 ± 1.78***	51.7 ± 2.30***	58.6 ± 1.57***	68.5 ± 2.34***	77.3 ± 1.90***	0.21 ± 0.0***	4.49 ± 0.14**
*F. globosum*									
MRC 6647	100 ± 2.84	15.1 ± 1.92***	25.2 ± 0.91***	26.5 ± 0.90***	52.5 ± 3.13***	71.9 ± 1.58***	77.7 ± 1.40***	27.1 ± 4.2***	2.62 ± 0.10**
MRC 6122	100 ± 1.99	11.3 ± 1.45***	17.0 ± 1.42***	21.1 ± 1.25***	33.6 ± 0.90***	40.5 ± 1.63***	47.7 ± 1.80***	24.5 ± 2.7***	0.70 ± 0.04**

a Values represent means ± standard deviations of five replications from at least two experiments. % ATP production, percentage of spore viability of cell energy production; IC_50_ of ATP, MIC at 50% ATP; *** and **, statistically significant at *P* < 0.0001, and *P* < 0.001, respectively.

**TABLE 5:** Conidial viability parameters after 96 h of exposure to unfermented *C. subternata* extract^*a*^

Fusarium strain	% ATP production								IC_50_ of ATP (mg/mL)
	Control	Unfermented *C. subternata* extract						AmpB (2.5 mg/mL)	
		20 mg/mL	10 mg/mL	5 mg/mL	2.5 mg/mL	1.25 mg/mL	0.62 mg/mL		
F. verticillioides									
MRC 826-E	100 ± 0.67	76.7 ± 0.94***	84.2 ± 1.91***	85.3 ± 1.76***	158.9 ± 8.5***	164.6 ± 11.8***	196.6 ± 13.1***	50.1 ± 4.3***	32.95 ± 0.6**
MRC 826-J	100 ± 1.11	30.6 ± 2.01***	34.0 ± 3.01***	53.2 ± 2.17***	56.6 ± 2.23***	70.9 ± 3.35***	86.4 ± 2.83***	23.3 ± 5.3***	4.83 ± 0.33**
MRC 8559	100 ± 1.35	23.9 ± 1.79***	25.4 ± 1.63***	59.5 ± 1.79***	78.1 ± 2.35***	88.5 ± 7.49***	110.4 ± 2.16***	2.08 ± 0.2***	6.23 ± 0.17**
*F. subglutinans*									
MRC 8554	100 ± 5.00	34.6 ± 2.24***	67.6 ± 2.93***	73.4 ± 0.84***	103.3 ± 3.8***	106.4 ± 1.95***	131.1 ± 7.21***	1.77 ± 0.3***	13.87 ± 0.39**
MRC 8553	100 ± 2.11	44.9 ± 2.14***	47.6 ± 0.84***	49.7 ± 1.72***	103.7 ± 1.8***	111.7 ± 7.00***	116.6 ± 2.47***	3.51 ± 0.1***	10.83 ± 0.47**
F. proliferatum									
MRC 8550	100 ± 3.19	87.1 ± 5.35***	87.7 ± 6.35***	99.9 ± 2.73***	116.0 ± 3.4***	109.6 ± 6.40***	143.9 ± 4.59***	44.1 ± 4.1***	45.88 ± 11.3**
MRC 8549	100 ± 3.34	24.9 ± 1.05***	56.1 ± 2.53***	82.9 ± 3.71***	96.4 ± 4.4***	114.8 ± 2.86***	138.9 ± 2.33***	45.2 ± 1.2***	11.33 ± 0.45**

F. graminearum MRC 4927	100 ± 2.84	47.8 ± 2.26***	63.4 ± 2.76***	72.4 ± 2.55***	111.3 ± 3.2***	116.6 ± 2.75***	127.6 ± 3.74***	0.21 ± 0.0***	17.17 ± 0.43**
*F. globosum*									
MRC 6647	100 ± 1.95	28.6 ± 1.54***	43.3 ± 2.36***	50.9 ± 2.02***	54.2 ± 2.4***	67.5 ± 3.17***	83.3 ± 2.08***	27.1 ± 4.2***	4.98 ± 0.23**
MRC 6122	100 ± 1.99	30.7 ± 1.28***	47.8 ± 1.34***	53.5 ± 1.69***	70.2 ± 1.17***	80.9 ± 2.41***	99.0 ± 1.61***	24.5 ± 2.7***	7.56 ± 0.32**

a Values represent means ± standard deviations of five replications from at least two experiments. % of ATP production, percentage of spore viability of cell energy production; IC_50_ of ATP, MIC at 50% ATP; *** and **, statistically significant at *P* < 0.0001 and *P* < 0.001, respectively.

**TABLE 6:** Conidia viability parameters after 96 h exposure to unfermented C. sinensis extract^*a*^

Fusarium strain	% ATP production								IC_50_ of ATP (mg/mL)
	Control	Unfermented C. sinensis extract						AmpB (2.5 mg/mL)	
		20 mg/mL	10 mg/mL	5 mg/mL	2.5 mg/mL	1.25 mg/mL	0.62 mg/mL		
F. verticillioides									
MRC 826-E	100 ± 0.67	56.4 ± 1.38***	122.6 ± 9.7***	150.0 ± 4.1***	160.6 ± 6.7***	176.9 ± 15.4***	196.0 ± 8.7***	50.1 ± 4.3***	0.00 ± 0.00**
MRC 826-J	100 ± 1.11	27.5 ± 1.29***	31.7 ± 1.07***	46.5 ± 2.86***	53.4 ± 1.37***	65.9 ± 1.03***	78.0 ± 1.17***	23.3 ± 5.3***	3.67 ± 0.21**
MRC 8559	100 ± 1.35	51.2 ± 1.48***	51.6 ± 1.51***	60.7 ± 2.48***	174.6 ± 17.6***	188.2 ± 8.7***	205.6 ± 12.3***	2.08 ± 0.2***	14.90 ± 0.46**
*F. subglutinans*									
MRC 8554	100 ± 5.00	121.5 ± 2.7***	291.6 ± 9.1***	399.9 ± 33.4***	406.7 ± 19.6***	439.4 ± 11.5***	480.9 ± 17.7***	1.77 ± 0.3***	0.00 ± 0.00**
MRC 8553	100 ± 2.11	77.2 ± 1.28***	94.7 ± 5.64***	115.1 ± 5.7***	168.8 ± 16.8***	188.2 ± 8.0***	241.2 ± 17.2***	3.51 ± 0.1***	27.40 ± 3.19**
F. proliferatum									
MRC 8550	100 ± 3.19	52.7 ± 2.48***	65.9 ± 6.23***	68.3 ± 4.59***	57.0 ± 8.58***	109.4 ± 15.5***	101.9 ± 1.0***	44.1 ± 4.1***	18.36 ± 5.36**
MRC 8549	100 ± 3.34	47.0 ± 1.52***	54.8 ± 2.28***	55.5 ± 2.27***	57.6 ± 2.48***	63.6 ± 2.14***	87.2 ± 1.03***	45.2 ± 1.2***	11.25 ± 1.42**

F. graminearum MRC 4927	100 ± 1.95	59.9 ± 2.25***	97.1 ± 15.1***	124.9 ± 3.3***	139.6 ± 3.6***	190.3 ± 15.0***	213.0 ± 17.3***	0.21 ± 0.0***	22.19 ± 0.93**
*F. globosum*									
MRC 6647	100 ± 2.84	25.0 ± 2.84***	42.8 ± 2.19***	61.7 ± 2.46***	120.7 ± 1.4***	139.6 ± 2.3***	161.2 ± 13.9***	27.1 ± 4.2***	9.26 ± 0.65**
MRC 6122	100 ± 1.99	43.9 ± 1.72***	59.6 ± 1.25***	80.6 ± 1.08***	87.8 ± 6.00***	93.8 ± 2.31***	103.8 ± 5.7***	24.5 ± 2.7***	15.23 ± 0.81**

a Values represent means ± standard deviations of five replications from at least two experiments. % ATP production, percentage of spore viability of cell energy production; IC_50_ of ATP, MIC at 50% ATP; *** and **, statistically significant at *P* < 0.0001 and *P* < 0.001, respectively.

**FIG 2:**
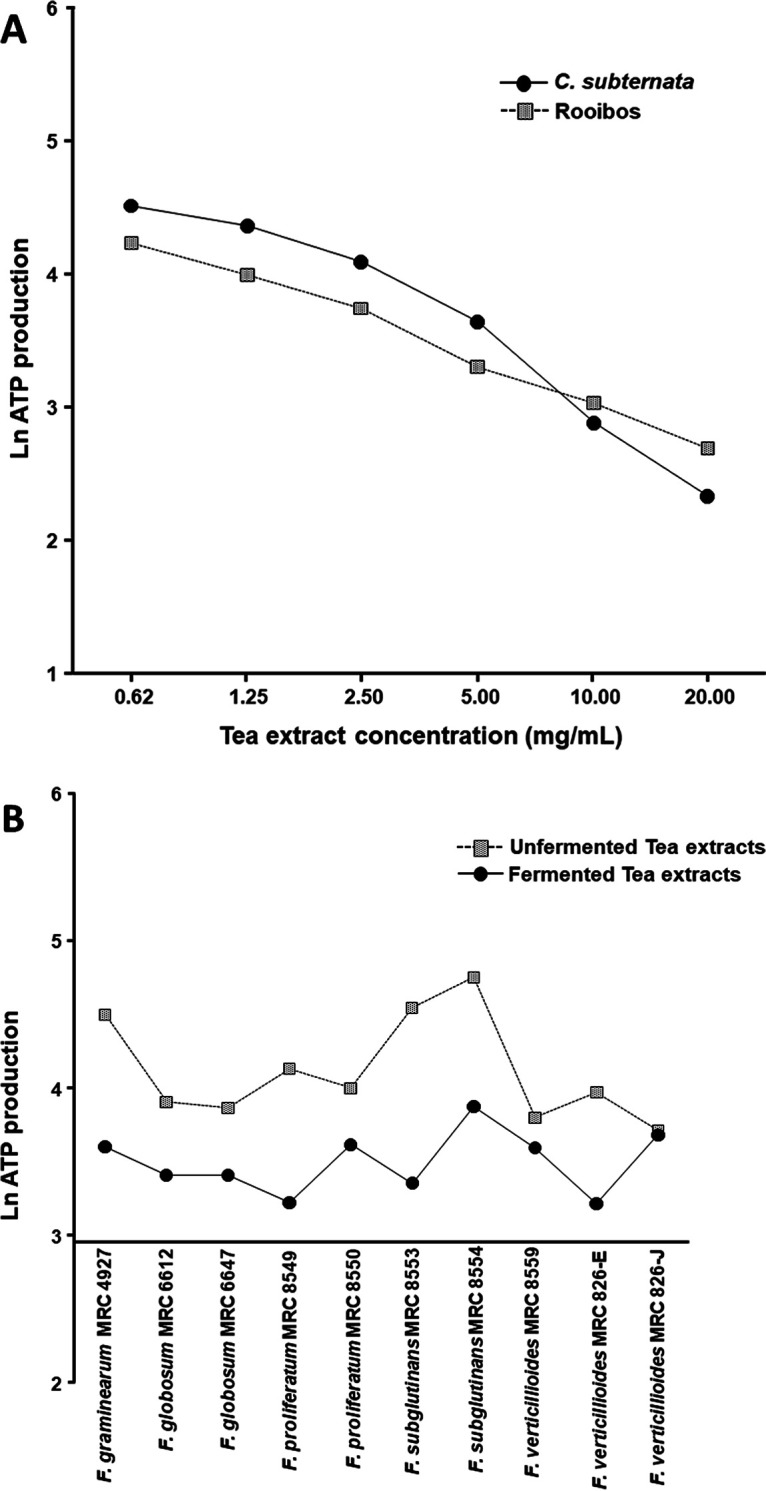
(A) Tukey-Kramer comparison plot of the unfermented and fermented rooibos combined and compared to unfermented and fermented *C. subternata*, indicating a significantly (*P* < 0.0001) higher growth inhibition rate for rooibos at the lower concentrations (0.62 to 5 mg/mL) for all Fusarium strains combined. (B) The comparison plot between the fermented and unfermented tea extracts (rooibos and honeybush) against all the Fusarium strains was also highly significant (*P* < 0.0001), showing the fermented tea extract to be the most active extract against conidial growth inhibition.

The comparison plot of the levels of rooibos and *C. subternata* growth inhibition against the various Fusarium strains at various concentrations was highly significant (*P* < 0.0001) (Fig. 2A), indicating a higher growth inhibition rate by rooibos extract. Furthermore, a comparison plot of the fermented and unfermented tea extracts against the Fusarium strains confirmed the fermented tea extracts to be more active (*P* < 0.0001) than the unfermented tea extracts (Fig. 2B). The single optimized dose (2.5 mg/mL) of amphotericin B (AmpB) used as an inhibitor control significantly (*P* < 0.0001) inhibited all spore growth of all 10 Fusarium strains, in a range of 0.21% to 50.1% (Table 2).

### Germination of Fusarium spores inhibited by tea extracts.

**FIG 3:**
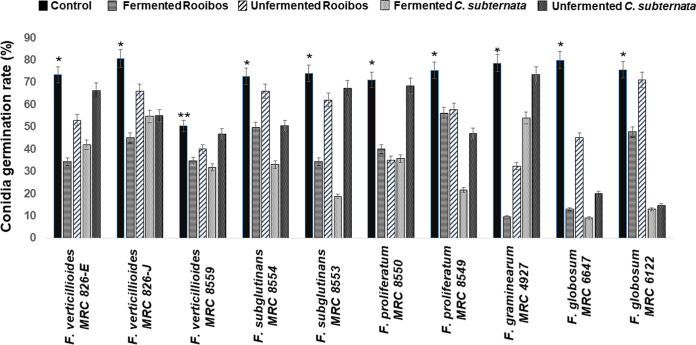
Fermented and unfermented tea extracts inhibit conidial germination of several Fusarium strains. Fusarium strain conidia were grown in a liquid medium without (control) or with 20 mg/mL tea extract and were incubated at 26°C for 24 h before microscopic observation at a magnification of ×400. The conidial germination rate was calculated by randomly counting 50 conidia from each of three independent experiments and then plotted using GraphPad Prism 5.01 software (GraphPad Software, Inc., La Jolla, CA). *, *P* ≤ 0.05, and **, *P* ≤ 0.01, for comparison between control and tea extracts for each species; #, *P* ≤ 0.05, and ##, *P* ≤ 0.01, for comparison between fermented and unfermented tea extracts for each species (e.g., fermented *C. subternata* versus unfermented *C. subternata*).

**FIG 4:**
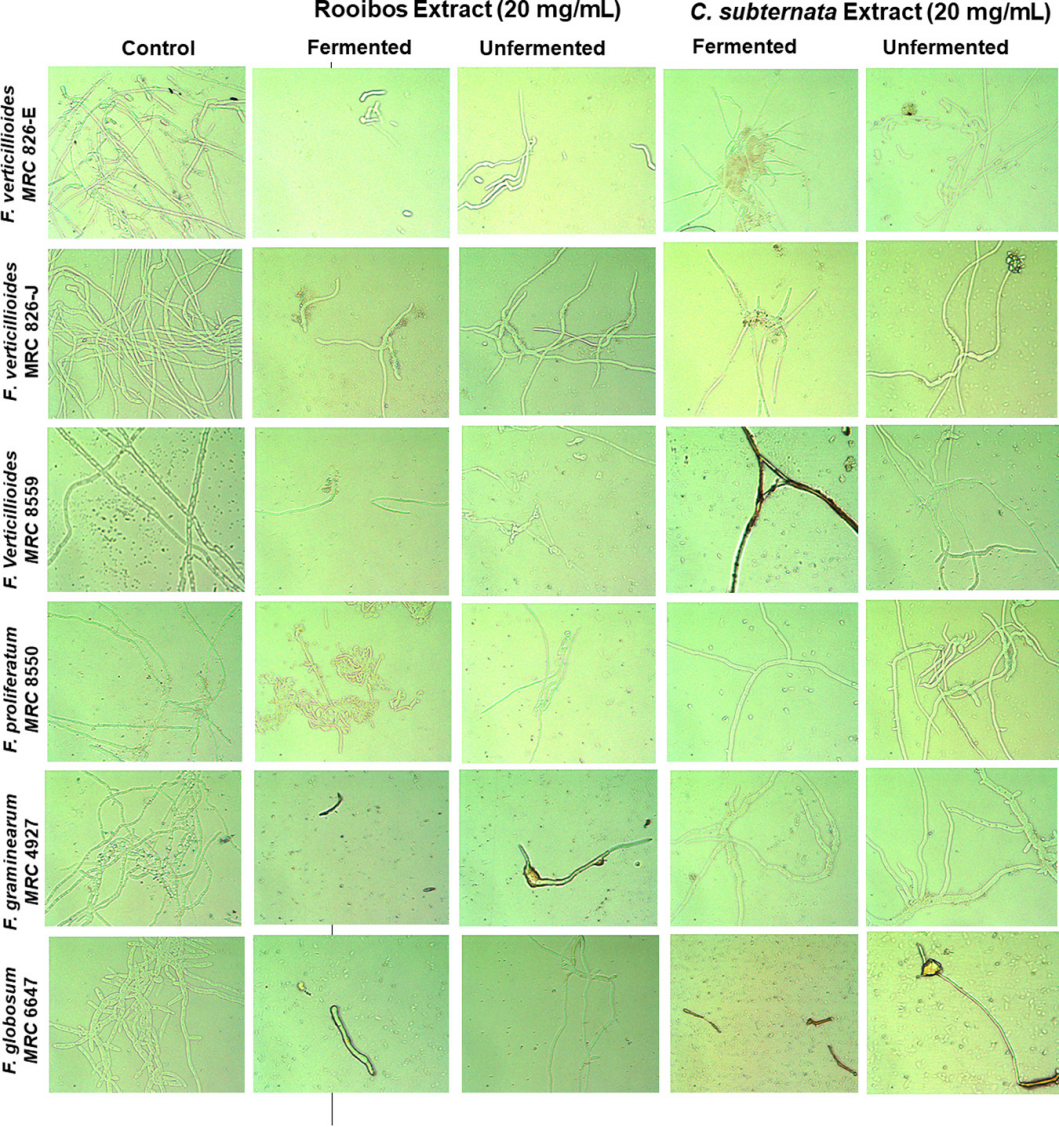
Formation of conidial germ tube inhibited with 20 mg/mL tea extract compared with the control. Microscopic observation was done at a magnification of ×400. Images were taken using the Zeiss MoticamPro 282A camera, with LED-Module/ex 640 nm detected in the 840/15 bandpass filter. Germination was defined as the presence of a thin germ tube longer than its respective conidium.

Spore germination of the Fusarium conidia exposed to each respective tea at 20 mg/mL showed inhibition of conidial germ tube formation of most Fusarium strains (Fig. 3 and Fig. 4). However, higher inhibition of conidia germination was observed with the fermented rooibos tea extract. With fermented *C. subternata* treatment, significant inhibition of conidial germination in *F. subglutinans* MRC 8553, F. proliferatum MRC 8549, *F. globosum* MRC 6647, and *F. globosum* MRC 6122 was observed. The unfermented rooibos extract showed less activity in reducing the germination rates of the Fusarium conidia. By inhibiting Fusarium conidial germination with tea extracts, the potential for infection progression can be reduced.

### Effect of herbal tea extracts on Fusarium spore morphology.

**FIG 5:**
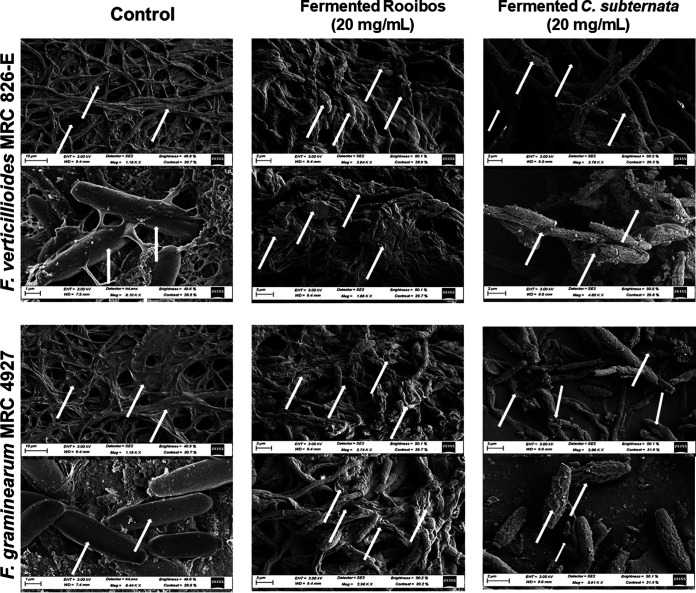
Scanning electron microscopy (SEM) observations of fungal hyphal morphological changes when exposed to teas. F. verticillioides MRC 826-E and F. graminearum MRC-4927 cells were incubated in a liquid medium without (control) or with 20 mg/mL tea extract and then submitted to SEM. After 96 h, untreated hyphae and conidia with regular cell walls are visible, as indicated by the arrows in the control samples. After treatment, both hyphae and conidia suffered from malformed structure swelling; arrows in the treated samples indicate ruptured hyphae and deformed conidia with flattening of cell membrane.

The effect of tea extracts on conidial spore morphology was determined using the two most resistant strains, including F. verticillioides MRC 826-E and F. graminearum MRC 4927. Treated conidial samples were examined using scanning electron microscopy (SEM) (Carl Zeiss Microscopy, Germany). Untreated (without tea) conidia on potato dextrose broth (PDB) medium incubated for 96 h at 26°C showed normal spore growth, with smooth linear hyphae that appeared regular in shape (Fig. 5). The conidial membrane of F. verticillioides MRC 826-E became highly affected after treatment with 20 mg/mL tea extract. High fungicidal disruption of the conidial membrane was observed in the samples treated with fermented rooibos and honeybush extracts. The fungicidal activity of fermented rooibos and honeybush extracts was also shown against F. graminearum MRC 4927. Hyphae of both Fusarium strains were collapsed, shrunk, and deformed, with a loss of linearity. Furthermore, craters were evident on both conidial cell membranes.

## DISCUSSION

Fusarium species are one of the most destructive fungal pathogens and cause severe postharvest contamination, especially in maize, wheat, soybean and sorghum. The present study investigated the antifungal effect of indigenous South African rooibos (*A. linearis*) and honeybush (*C. subternata*) tea and observed that the tea extracts significantly inhibited the conidial growth of several Fusarium species. The fermented rooibos extract was 2-fold more active in growth inhibition than the unfermented rooibos extract. Comparing the activity of honeybush extracts against the Fusarium conidial growth inhibition, the fermented *C. subternata* extract proved more active than the unfermented *C. subternata*. The antifungal activity of unfermented C. sinensis was 2-fold less than those of rooibos and honeybush tea extracts. Many studies have evaluated the antimicrobial effects of rooibos and honeybush teas (13, 14, 16, 17). Exposure of *B. cinerea* spores to rooibos and honeybush (Cyclopia genistoides) tea extracts at 100 mg/mL inhibited growth by 33% and 16%, respectively (14). The rooibos and honeybush tea extracts, including *C. subternata* tested on E. coli used as a benchmark, were observed to inhibit cell growth by 60%, 80%, and 85%, respectively, after 6 h. Similar results were noted for both Staphylococcus aureus and Staphylococcus epidermis, with fermented rooibos being more active than unfermented rooibos by 85% versus 35% and 78% versus 23%, respectively (17). The minimum inhibitory activity of the fermented honeybush *C. intermedia* aqueous extract was shown to reduce the cell growth of C. albicans at 150 mg/mL (13). Thembo et al. (18) also reported the antifungal inhibitory effects of four South African weedy aqueous extracts against F. verticillioides, F. proliferatum, Aspergillus flavus, and Aspergillus parasiticus at a concentration of 2.5 mg/mL. However, the results in the study by Thembo et al. (18) showed little or no inhibitory effect due to the low concentration they used compared to the high concentration (20 mg/mL) used in this study.

At the lower concentrations (5 mg/mL and lower), the rooibos and *C. subternata* extracts in this study increased the ATP percentage above 100% for most treatments after 96 h. This phenomenon could indicate fungal growth stimulation at lower tea concentrations. Other possible reasons could be that the antioxidant components present in the teas may no longer be effective enough to inhibit the conidial growth *per se* but rather stimulate the cell biomass (19). Simpson et al. (17) also recorded a similar effect for E. coli, where the maximum optical density (OD_max_) values for all concentrations below 10 μg GAE (gallic acid equivalent) for both rooibos infusions and unfermented rooibos were contrary to expectation, as bacterial growth was enhanced rather than suppressed after 6 h. Their most reasonable explanation for the outcome was probably the inherent nutritional value, combined with reduced toxicity at lower concentrations. Coetzee et al. (14) observed *B. cinerea* cells in a low concentration (10 mg/mL) of rooibos and *C. subternata* extracts and noticed induced biomass production by more than 2- to 3-fold after 48 h. Another hypothesis could be that the compounds act synergistically in inhibiting growth, and at lower concentrations, one of the compounds may be diluted out, affecting the synergistic effect. It is also possible that glycosides are present at a higher concentration and when a diluted extract is used, the glycosides are still present, acting as an additional carbon source for the fungus. Third, a lower tea extract concentration could also cause growth stimulation due to some of the vital mineral nutrients of potassium (K), calcium (Ca), magnesium (Mg), and manganese (Mn) present in the PDB growth medium combined with the tea's nutrients (Ca, K, Mg, and Na) (14). Therefore, the concentration of tea extracts is of utmost importance when considering antimicrobial inhibition studies.

A 20-mg/mL treatment with fermented rooibos extract significantly reduced the germination of spores in F. verticillioides MRC 826-E, MRC 826-J, and MRC 8559, F. graminearum MRC 4927, and *F. globosum* MRC 6647 compared to the other teas. From the results obtained in the study, it would be difficult to point out exactly which group of compounds were responsible for the significant effect. In an antimicrobial study by Simpson et al. (17), the polyphenols from both naturally fermented rooibos and artificial infusions were individually tested at the highest concentrations (2.0 and 4.5 μg) on E. coli (strain CFT073), S. epidermis (strain Se19), and S. aureus (strain ATCC 25923). However, no changes in bacterial growth inhibition were observed compared to the control medium only (17). They concluded that the glycosylated polyphenols alone might not account for the inhibition observed when testing the natural extracts. Therefore, bacterial uptake of several polyphenols from crude extracts could have reduced the cell viability or can even represent the presence of unidentified polyphenols with antibacterial activity. We speculate that the polyphenols—most likely the glycosylated compounds present in rooibos extracts, including *S*-eriodictyol-6-glucoside (E6CG), *S*-eriodictyol-8-glucoside (E8CG), phenylpyruvic acid-2-*O*-glucoside (PPAG), aspalathin, isoorientin, orientin, hyperoside, rutin, isoquercitrin, luteoloside, nothofagin, vitexin, and isovitexin—as well as the nonglycosylated bioquercetin may have reduced the conidial cell viability. The majority of these polyphenols have been shown to be active against one or more inflammatory infections. For example, the flavanone glycosides and eriodictyols in fermented rooibos have been reported to have natural antioxidant and anti-inflammatory, antidiabetes, antiobesity, and skin protection activities (20).

The polyphenolic contents of the *C. subternata* extracts were distinct from those in C. sinensis, being caffeine free with low levels of tannin. The main polyphenols found include xanthones, flavones, isoflavones, flavanones, flavanols, and coumestans. Contrary to previous studies (21, 22), the fermented *C. subternata* polyphenols showed improved antifungal activity compared to those in unfermented extracts, even though the total amount of polyphenols was larger (9.105 g/100 g extract) in unfermented than in fermented (4.333 g/100 g extract) extract. Reasons for this effect are not widely reported; however, available evidence has reported a wide range of pharmacological activities of C-glycosyl mangiferin, hesperidin, xanthone, and scolymoside (23). Hesperidin was recently shown to bind to COVID-19-spike protein and interfere with the refolding of the spike, inhibiting viral infection (24). The antimicrobial activities of tea dihydrochalcones (phloretin and its glycosylated derivatives) tested against several clinical Gram-positive and Gram-negative bacterial strains were observed to inhibit the growth of S. aureus ATCC 6538, Salmonella enterica serovar Typhimurium ATCC 13311, Listeria monocytogenes ATCC 13932, and methicillin-resistant S. aureus clinical strains from 7.81 μg/mL to >1,000 μg/mL (25). The polyphenols analyzed in C. sinensis were mainly caffeine, followed by epigallocatechins. According to Coetzee et al. (14), the antimicrobial effect of C. sinensis was directly linked to the presence of epicatechin gallate and epigallocatechin gallate. These compounds were found to have an inhibitory effect on methicillin-resistant S. aureus, multidrug-resistant Pseudomonas aeruginosa, E. coli, Enterococcus faecalis, and C. albicans. This suggests that the antifungal property of the tested tea extracts may be due to these polyphenols.

The mode of action of many antimicrobial agents analyzed by SEM targets the biosynthesis of ergosterol by disrupting either cell envelope integrity or biofilm formation (26). In this study, F. verticillioides MRC 826-E and F. graminearum MRC 4927 damaged conidial cell envelope was visible before and after tea treatment at 20 mg/mL with both the fermented rooibos and *C. subternata* extracts. The disruption of the cell membranes may have resulted in the osmotic imbalance of the mycelial enzymatic reactions, thus preventing further morphogenesis and growth development of the fungi (27).

### Conclusion.

This is the first study to investigate the use and efficacy of extracts from *A. linearis* and *C. subternata* as innovative antimicrobial agents against Fusarium species. The fermented rooibos and honeybush tea extracts demonstrated higher antifungal susceptibility than the unfermented tea extracts. The effect could be the combination of several polyphenols present in the tea extracts, which may have acted on the cell envelope. The antifungal activity of the tea extracts was shown against several Fusarium strains that are known to produce mycotoxins. If the extracts could suppress conidial viability or prevent conidial germination, it could prevent the production of these mycotoxins. However, more research is required to understand the underlying mode of action and develop suitable, sustainable, effective, inexpensive botanical products that can be used to help overcome the scourge of plant fungal diseases, which could lead to increased food safety and security.

## MATERIALS AND METHODS

### Chemicals.

The chemicals used in this study were of analytical grade, purchased from Sigma-Aldrich (Merck, USA), including dimethyl sulfoxide (DMSO), sucrose, magnesium sulfate (MgSO_4_·7H_2_O), potassium chloride (KCl), potassium dihydrogen phosphate (KH_2_PO_4_), calcium nitrate [Ca(NO_3_)_2_], ferric chloride (FeCl_3_), manganese sulfate (MnSO_4_), zinc sulfate (ZnSO_4_), Hoechst 33342, and propidium iodide, as well as amphotericin B. BacTiter-Glo luminescent microbial cell viability assay kits were obtained from Anatech (Promega, Madison, WI, USA).

### Preparation and characterization of herbal tea extracts.

Fermented and unfermented rooibos (*A. linearis*) and honeybush (*C. subternata*) tea were obtained from the ARC-Infruitec (Agriculture Research Council), Stellenbosch, South Africa. The green tea (Camellia sinensis), imported from China, was a gift from Vital Health Foods, Kuilsriver, South Africa. Aqueous extracts were prepared by adding 1 L of freshly boiled distilled water to 100 g plant material in a 2-L conical flask and then incubating the mixtures for 30 min at 22 ± 3°C, stirring every 5 min. The extracts were strained through a double layer of cheesecloth to remove the majority of the plant material. An additional filtration was performed using Whatman no. 1 filter paper (Merck, Darmstadt, Germany). The filtrates were lyophilized, and the final percentages of each tea were calculated as follows, where *X* is the mass of each tea material (grams), *A* is the mass of the empty round-bottom flask (grams), and *B* is the mass of the round-bottom flask plus tea extracted (grams): mass of tea (g) = (*B* − *A*) and % yield = [(*B* − *A*)/*X*] × 100. All dried extracts were stored in amber bottles and preserved in a desiccator at 22 ± 3°C prior to use. HPLC-DAD quantification of the individual polyphenols in the extracts was analyzed by the ARC-Infruitec South Africa (15).

### Fungal strains and culture conditions.

The Fusarium strains used in this study are shown in Table 7. The strains were obtained from the Applied Microbial and Health Biotechnology Institute (AMHBI) culture collection and stored at 4°C as lyophilized spore suspensions.

**TABLE 7:** Fusarium strains used in this study

Fusarium species	MRC isolate	Reference or source
F. verticillioides	MRC 826-E	30
	MRC 826-J	30
	MRC 8559	Isolated from *s*orghum stalk in CA, USA, in January 2004

F. graminearum	MRC 4927	Isolated from wheat ear scab at Stander Farm, in Pacaltsdorp, South Africa, in October 1987

F. subglutinans	MRC 8553	Isolated from corn in St. Elmo, IL, USA, in January 2004^*a*^
	MRC 8554	Isolated from corn in St. Elmo, IL, USA, in January 2004^*a*^

F. proliferatum	MRC 8549	Progeny of cross (corn × sorghum isolates) 00502 × 02945 1013G5 in January 2004^*a*^
	MRC 8550	Progeny of cross (corn × sorghum isolates) 00502 × 02945 1013G33 in January 2004^*a*^

F. globosum	MRC 6647	Isolated from maize in Eastern Cape, South Africa, in September 1992 (31)
	MRC 6122	Isolated from sorghum soil debris, Eastern Cape, South Africa, in September 1992

a Received from John Leslie.

Rehydrated freeze-dried spore suspensions (1 mL distilled water [dH_2_O]) of the different MRC strains were inoculated onto potato dextrose agar (PDA) plates and incubated at 26°C for 2 weeks. For sporulation, Armstrong medium (28) was inoculated with the different Fusarium strains from the PDA plates in Erlenmeyer flasks (250 mL) and incubated in a shaker incubator (New Brunswick) at 100 rpm at 26°C for 5 days. Mycelial growth was removed by filtration of the suspensions through a double layer of sterile cheesecloth. Conidial suspensions were transferred to 50-mL sterile Falcon tubes and centrifuged at 4,000 rpm for 10 min, and the supernatant was removed. Conidia were washed twice with sterile deionized water of volumes equivalent to that of the original suspension. Conidia were counted using a Neubauer hemocytometer, diluted to 1 × 10^6^ conidia/mL, and stored at 4°C.

Conidial viability (200 μL) was determined utilizing 1 μL Hoechst 33342 (Sigma-Aldrich) and 2 μL propidium iodide (Sigma-Aldrich) stain. The reaction mixture was incubated for 10 min at 22 ± 3°C, and 100 μL of the stained samples was pipetted into an 8-well chamber (Thermo Scientific). Samples were viewed using a 20× lens objective (LD A-plan 20×/0.35 Ph2), and images were taken using the Zeiss Axiocam 503 mono camera, with the LED-Module/ex 511-nm/bandpass filter 555/25 for propidium iodide and the LED-Module/Ex385nm/425/30 bandpass filter for Hoechst 33342. ZEN 2.6 (blue edition) software was used to acquire and process the images.

### Evaluation of antifungal inhibition of the tea extracts on ATP production.

The effect of the different concentrations of the tea extracts on conidial viability was evaluated using the BacTiter-Glo cell viability reagent (ADG8232; Promega, USA) to measure ATP production. Briefly, 20 mg of each tea extract was dissolved in 1 mL potato dextrose broth (PDB) (Merck, Darmstadt, Germany) containing 1% (vol/vol) DMSO. Serial dilutions were prepared to obtain the final concentrations ranging from 20 mg/mL to 0.625 mg/mL. Fifty microliters of each tea extract was dispensed into the respective 96-well plates (white Falcon opaque plates with a low-evaporation lid) (1060 LJ; Amsterdam, Netherlands) with 5 replicates per treatment. Fifty microliters of the conidial suspension (density of 1 × 10^6^ conidia/mL) was added to each well. Fifty microliters of only broth medium and 50 μL conidial suspension (density of 1 × 10^6^ conidia/mL) without tea served as negative controls. Amphotericin B (2.5 mg/mL) was used as a positive control for antifungal activity (29). Plates were sealed with parafilm and incubated for 96 h at 26°C. BacTiter-Glo microbial cell viability reagent (100 μL) was added to all wells after incubation. The plate was shaken briefly for 2 min and allowed to incubate in the dark for an additional 2 min. ATP production was measured using the Veritas 96 microplate luminometer (Turner BioSystems, Inc., Sunnyvale, CA, USA). The extracts’ MICs were determined using GraphPad Prism 5.01 software (GraphPad Software, Inc., La Jolla, CA).

### Evaluation of the antifungal activity of the tea extracts on growth inhibition.

Fusarium conidia (1 × 10^6^ conidia/mL) were added to the respective 20-mg/mL tea extracts in Difco PDB (Merck, Darmstadt, Germany) in a final volume of 200 μL. Fusarium conidia (1 × 10^6^ conidia/mL) in PDB in a final volume of 200 μL were used as the negative control. Cultures were incubated for 24 h at 26°C in the dark to minimize oxidation of the tea extracts. Following incubation, the supernatant was removed by centrifugation (4,000 rpm for 10 min), and the pellet was resuspended in 2% (vol/vol) Tween 20 (Polysorbate 20; Sigma-Aldrich). Conidial germination was observed utilizing a fluorescence electron microscope (Zeiss MoticamPro; Nikon-YFL-080417). Images were taken using the Zeiss MoticamPro 282A camera, with LED-Module/ex 640 nm detected in the 840/15 bandpass filter. Germination was observed when a thin germ tube was longer than its respective conidium. The germination rate was calculated by randomly counting 50 conidia from each of three independent experiments. To compare conidial germination rates between groups, statistical analysis (Student's unpaired *t* test) was conducted using GraphPad Prism 5.01 software (GraphPad Software, Inc., La Jolla, CA). Statistical significance was set at a *P* value of <0.05.

### Scanning electron microscopy.

The Fusarium conidia treated with the herbal tea extracts as described above were fixed in 2% (vol/vol) glutaraldehyde with 4% (vol/vol) paraformaldehyde (PFA) in 0.1 M Na-cacodylate buffer (pH 7.3) at 4°C for 4 h. Cells were allowed to settle onto poly-l-lysine-coated 12-mm coverslips. To prevent drying, the cells were fixed *in situ* in the same primary fixative, using a multiwall plate/quad-petri-dish and covered. Postfixation, the cells were briefly washed twice in 0.1 M Na-cacodylate containing 1% (vol/vol) aqueous OsO_4_ (1 h at 4°C), washed 3 times in dH_2_O, and then dehydrated in a series of graded (50% to 70% to 90% [vol/vol]) ethanol and subjected to drying with a head-mounted display (HMDS) infiltration unit. Samples were mounted onto a 12-mm aluminum SEM stub using double-sided carbon conductive tape and coated with 50 to 100-Å Au/Pd using a sputter coater (Leica EM ACE200) for 3 min and visualized and identified at different magnifications under a scanning electron microscope (Carl Zeiss Microscopy, Germany). Images were captured using SmartSEM (Zeiss Electron Imaging) and Oxford AZtec (Oxford Instruments, Abingdon, Oxfordshire, United Kingdom).

### Statistical analysis of data.

The data were analyzed using NCSS 2021 v.21.0.2. Data were subjected to natural log (ln) transformation of all variables and analyzed within a generalized linear model analysis of variance (ANOVA). Multiple comparisons were analyzed using the Tukey-Kramer multiple-comparison procedure. This method provides joint simultaneous confidence intervals for all pairwise differences between the means as well as provides the multiple comparison *P* value. Overall, a *P* value of <0.05 was used to represent statistical significance. In addition, the size of the *F* ratios was used to measure relative sizes of differences.
